# A Maximum Entropy Test for Evaluating Higher-Order Correlations in Spike Counts

**DOI:** 10.1371/journal.pcbi.1002539

**Published:** 2012-06-07

**Authors:** Arno Onken, Valentin Dragoi, Klaus Obermayer

**Affiliations:** 1 Technische Universität Berlin, Berlin, Germany; 2 Bernstein Center for Computational Neuroscience Berlin, Berlin, Germany; 3 University of Texas, Houston Medical School, Houston, Texas, United States of America; MIT, United States of America

## Abstract

Evaluating the importance of higher-order correlations of neural spike counts has been notoriously hard. A large number of samples are typically required in order to estimate higher-order correlations and resulting information theoretic quantities. In typical electrophysiology data sets with many experimental conditions, however, the number of samples in each condition is rather small. Here we describe a method that allows to quantify evidence for higher-order correlations in exactly these cases. We construct a family of reference distributions: maximum entropy distributions, which are constrained only by marginals and by linear correlations as quantified by the Pearson correlation coefficient. We devise a Monte Carlo goodness-of-fit test, which tests - for a given divergence measure of interest - whether the experimental data lead to the rejection of the null hypothesis that it was generated by one of the reference distributions. Applying our test to artificial data shows that the effects of higher-order correlations on these divergence measures can be detected even when the number of samples is small. Subsequently, we apply our method to spike count data which were recorded with multielectrode arrays from the primary visual cortex of anesthetized cat during an adaptation experiment. Using mutual information as a divergence measure we find that there are spike count bin sizes at which the maximum entropy hypothesis can be rejected for a substantial number of neuronal pairs. These results demonstrate that higher-order correlations can matter when estimating information theoretic quantities in V1. They also show that our test is able to detect their presence in typical in-vivo data sets, where the number of samples is too small to estimate higher-order correlations directly.

## Author Summary

Populations of neurons signal information by their joint activity. Dependencies between the activity of multiple neurons are typically described by the linear correlation coefficient. However, this description of the dependencies is not complete. Dependencies beyond the linear correlation coefficient, so-called higher-order correlations, are often neglected because too many experimental samples are required in order to estimate them reliably. Evaluating the importance of higher-order correlations for the neural representation has therefore been notoriously hard. We devise a statistical test that can quantify evidence for higher-order correlations without estimating higher-order correlations directly. The test yields reliable results even when the number of experimental samples is small. The power of the method is demonstrated on data which were recorded from a population of neurons in the primary visual cortex of cat during an adaptation experiment. We show that higher-order correlations can have a substantial impact on the encoded stimulus information which, moreover, is modulated by stimulus adaptation.

## Introduction

Neural coding examines the way that populations of neurons represent and signal information. A central topic in population coding is the impact of spike count correlations following repeated presentation of the same stimulus (noise correlations). How important are these dependencies for the information carried by the neural population response? Is it important for a decoder to take noise correlations into account? Typically, a distinction is made between (easy to estimate) linear and (hard to estimate) higher-order correlations. We define higher-order correlations as a statistical moment of order greater than two that is not uniquely determined by the first- and second-order statistics. According to this definition, higher-order correlations go beyond the linear correlation and can - but not necessarily have to - involve more than two neurons. These higher-order correlations can exist between pairs of neurons. They refer to all dependencies that are not already characterized by the correlation coefficients.

The linear correlation coefficient is central to many studies dealing with neural coding. Substantial correlations were found in many cortical areas (see e.g. [1]–[3]) with a notable exception being reported in [4]. Furthermore, characteristics of linear noise correlations change with adaptation [1]. Theoretical studies have revealed a strong impact of linear correlations on information measures and optimal decoding [5]–[12]. However, these studies have focused exclusively on linear correlations.

Higher-order correlations are notoriously difficult to estimate. The number of samples required for reliable estimation increases exponentially with the order of correlations [13]. Recently, statistical tests were developed for detection of higher-order correlations and cumulants [13], [14]. These tests are based on the compound Poisson process as an underlying model and may fail if the model assumptions are not justified. Furthermore, the tests were designed to *detect* higher-order dependencies and not to evaluate their impact on a neural coding measure of interest.

In order to *assess* the impact of correlations on neural coding, a performance measure must be evaluated which quantifies the “quality” of the neural code. Common measures include the decoding error (e.g. the averaged error of an optimal estimator), the Fisher information (for continuous variables), or the mutual information [15]. Calculation of most of these measures, however, requires full knowledge of the probability distribution of the data.

Probability distributions can be estimated without parametric assumptions using histograms. The histograms can then be used to estimate information theoretic quantities such as the mutual information. However, estimators based on histograms are biased if the sample size is small [16]. Bias correction techniques have been developed for alleviating this problem [16]–[18]. Breaking down the mutual information into several blocks that can be attributed to different statistics of the distribution can also help in reducing the number of required samples [11], [19], [20]. Nevertheless, the number of samples required for non-parametric estimators is still on the order of hundreds.

A complementary approach to estimate probability distributions involves model-based techniques with parametrized higher-order correlations. The most common way to analyze the impact of higher-order correlations is to fit a second-order model (which is based on first- and second-order statistics) to the data and to make a comparison to a higher-order model [21]–[29]. The distribution is typically assumed to be stationary over time. The method is restricted to situations in which it is possible to collect a sufficient number of samples for all stimulus conditions. For retinal ganglion cells, for instance, between 100 and 1000 samples can be collected for each bin of the neural activity distribution [29].

Maximum entropy distributions form a family of parametric models that allow to reliably quantify the impact of linear correlations [17], [30], because single neuron distributions (marginal distributions) and the linear correlation coefficient can be estimated reliably given a small number of samples. The model distribution can then be used to determine above mentioned measures quantifying the quality of the neural code. In principle, maximum entropy methods allow for the inclusion of higher-order correlations also [24]. However, these correlations have to be estimated from the data in order to be included as proper constraints requiring larger amounts of data. Ignoring these higher-order correlations, on the other hand, could possibly lead to biased results. Rich parametric families of distributions, such as copulas, reduce the number of required samples as a trade-off for parametric assumptions [23], [31]. Nevertheless, the number of samples that is required to reliably estimate the model parameters is still on the order of hundreds. In summary, previously described methods for detecting and evaluating the impact of higher-order correlations exhibit a number of shortcomings. They either (1) detect higher-order correlations without evaluating their impact on a neural coding measure; (2) are based on strong parametric assumptions; or (3) require a substantial number of samples to construct models that explicitly take higher-order correlations into account, which can be time-consuming and expensive to obtain. In these situations, it would be important to develop methods that assess, based on small sample sizes, whether higher-order correlations may have an impact on the conclusion to be drawn from a particular study or whether a collection of a large number of samples would be indeed required. For instance, let us assume that mutual information between an ensemble of stimuli and the responses of a small population of simultaneously recorded neurons is evaluated. It would then be desirable to design a test based on a small number of samples to assess whether higher-order correlations are present and they lead to conclusions substantially different from the conclusions obtained using linear correlations only.

Here, we introduce a statistical test to assess whether linear correlations are sufficient for analyzing population spike counts (null hypothesis). To this end, we construct a set of distributions which includes all maximum entropy distributions with linear correlations and a parametric family of marginals and test whether the data is consistent with or rejects the null hypothesis for the selected divergence measures.

This test is applied to all neuronal pairs for a given population. Hence, the test is sensitive to higher-order correlations between pairs only. The null hypothesis cannot be rejected if all distributions for the pairwise elements are consistent with the maximum entropy distribution. If, however, the null hypothesis is rejected for some of the pairs we can conclude that higher-order correlations are essential and need to be determined using a larger number of samples.

The paper is organized as follows. The next section contains a detailed description of the statistical test for maximum entropy distributions and the recording procedures. In Section “Results” we verify the statistical test for maximum entropy distributions on various dependency structures that were artificially generated. We then describe the results of the application to recordings from cat V1. The paper concludes with a discussion of the advantages and limitations of the approach and of the findings in V1.

## Materials and Methods

### Ethics Statement

All experiments were performed under protocols approved by MITs Animal Care and Use Committee.

### A Monte Carlo Maximum Entropy Test

Here we describe a novel test for bivariate spike count distributions that determines whether the dependence structure is sufficiently well characterized by the correlation coefficient. We will first give an intuitive description of the test followed by a rigorous mathematical description.

The test consists of two parts: (1) construction of a reference distribution which is based on the single neuron spike count distributions and the correlation coefficient and (2) a goodness-of-fit test to calculate a *p*-value and eventually reject the reference distribution.

The reference distribution formalizes the linear dependency assumption. For this purpose, we apply a maximum entropy model subject to a set of constraints. The constraints contain the complete single neuron spike count distributions and the linear correlation coefficient. Everything is therefore fixed by the distribution constraints except for the higher-order correlations. If this reference distribution can be statistically rejected then we can conclude that higher-order correlations do matter.

The single neuron spike count distributions and the correlation coefficient are not known a priori. Instead, they must be estimated from the data. For simplicity, we assume that the single neuron distributions are Poisson distributed. This leaves us with the estimation of firing rates and the correlation coefficient. The test should be applicable even when the number of samples is very small. Therefore, any estimates of distribution parameters are not reliable. Instead of relying on specific estimates of these parameters, we maximize the *p*-value over these parameters and then use the most conservative *p*-value.

**Figure 1:**
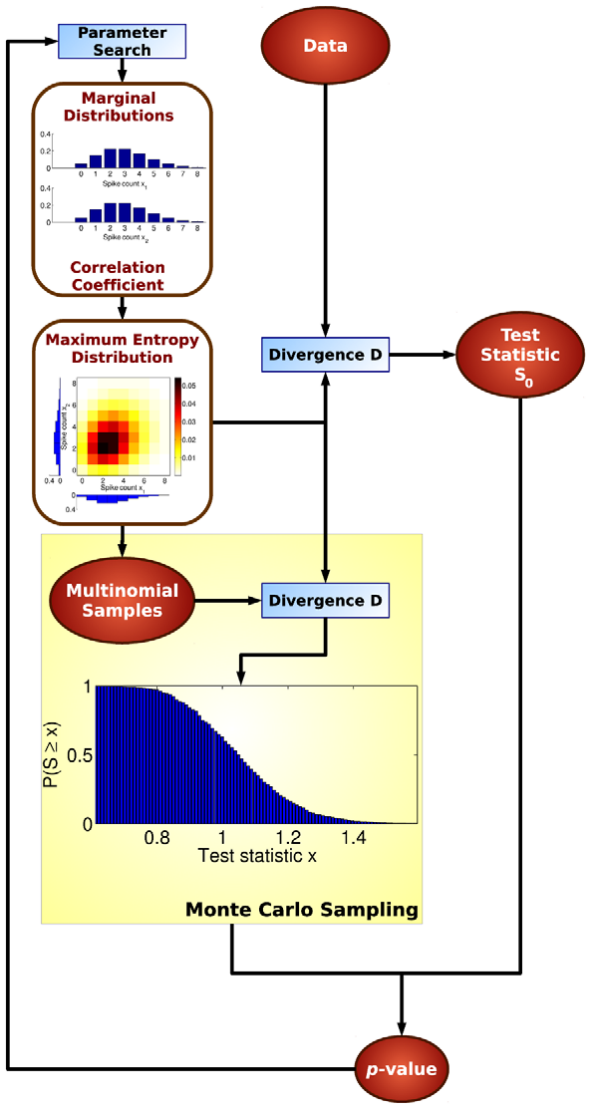
Flow diagram of the maximum entropy test.

Figure 1 shows a flow diagram of the test. A step-by-step procedure is provided in Table 1. In step (1) the Poisson parameters and the correlation coefficient are initialized with their sample means that are obtained from the data set. The *p*-value is then maximized by applying an optimization algorithm like simulated annealing (step (2), cf. Section “Optimization of the Nuisance Parameters” in Text S1). To this end, we estimate the *p*-value based on the maximum entropy distribution subject to the optimization parameters. First, we calculate a divergence 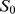 between the data and the maximum entropy distribution (step 2.2). We then draw many samples from the maximum entropy distribution and estimate an empirical distribution over divergences (step 2.3). By comparing the divergence 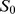 to this empirical distribution we can assess how likely it is that the maximum entropy distribution generated the data. This gives us a *p*-value for a particular set of Poisson rates and a correlation coefficient (step 2.4). The maximization over these parameters then yields the most conservative *p*-value. In step (3) the second-order assumption is rejected if the *p*-value is below the 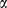 significance level.

**Table 1:** Step procedure of the Monte Carlo Maximum Entropy Test.

(1) Initialize spike count rates 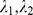 and correlation coefficient 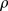 with their sample estimates from the data set 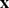
(2) Maximize the *p*-value over 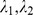 and 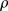 using simulated annealing:
(2.1) Compute the maximum entropy distribution 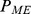 subject to Poisson marginals with rates 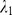 and 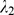 and correlation 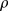
(2.2) Calculate the divergence 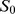 between the empirical distribution of the data set 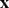 and the maximum entropy distribution 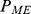
(2.3) For 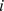 in 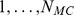 :
(2.3.1) Draw 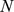 samples from 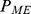 , where 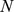 is the number of samples in the data set 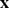
(2.3.2) Calculate the divergence 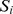 between the empirical distribution of the 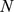 drawn samples and the maximum entropy distribution 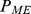
(2.4) Estimate the *p*-value based on the number of indices 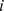 for which 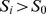 ; if 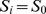 then toss a coin to decide whether to include the index
(3) Reject 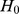 if 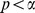

An implementation of the test including an application scenario for MathWorks MATLAB and GNU Octave is available online at a software directory of the Technische Universität Berlin (http://www.ni.tu-berlin.de/menue/software/monte_carlo_maximum_entropy_test/).

#### Formal test description

We will first describe the maximum entropy reference distribution and then the goodness-of-fit test based on a Monte Carlo procedure.

Consider a bivariate distribution 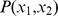 over 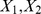 with marginals
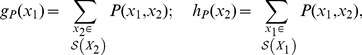
(1)where 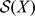 denotes the support of the random variable 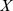. The linear correlation coefficient is given by
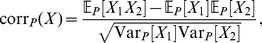
(2)where 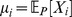 denotes the expectation and 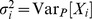 denotes the variance of 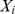. The maximum entropy distribution constrained by the marginals (Equation 1) of 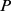 and the correlation coefficient (Equation 2) is the distribution 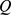 that maximizes the entropy 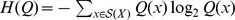 subject to the constraints:
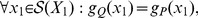
(3)

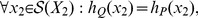
(4)

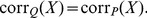
(5)It was shown [32] that this distribution is uniquely given by
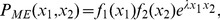
(6)where 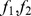 and 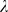 are obtained by solving:
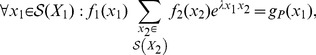
(7)

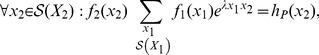
(8)

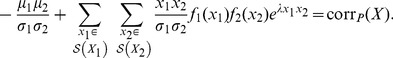
(9)


In order to examine whether a given data set is consistent with such a maximum entropy reference distribution we apply the multinomial distribution. Every bivariate discrete distribution with finite support can be represented by a table containing the probability distribution mass function. Let 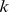 denote the number of boxes of this table with frequencies 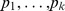. For a given number of draws 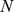 from this distribution the probability mass function of these entire draws follows a multinomial distribution:
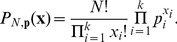
(10)The number of draws, 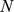, corresponds to the number of observations in the data set. The maximum entropy distributions constrained up to second-order constitute a subset 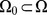 of the set 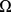 of all multinomial distributions. Let 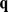 denote the frequencies of the true distribution of the observed data. We then consider the null hypothesis 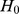, that the observed data are drawn from a distribution from 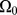:
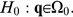
(11)If the null hypothesis can be rejected, then the marginals and linear correlation coefficient are not sufficient to characterize the underlying distribution of the data. This is formalized as the alternative hypothesis:
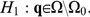
(12)


In order to test the null hypothesis the distributions of the test statistics are approximated by Monte Carlo sampling over nuisance parameters. The nuisance parameters 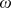 are unknown parameters such as the correlation coefficient or parameters of the marginal distributions. The Monte Carlo goodness-of-fit test with nuisance parameters follows [33]: for a given set of nuisance parameters and resulting reference distribution (in our case the maximum entropy distribution), Monte Carlo samples are drawn. A single Monte Carlo sample consists of 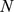 samples 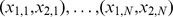 from the reference distribution. A total of 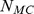 Monte Carlo samples 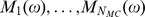 are drawn. Each Monte Carlo sample gives rise to an empirical distribution
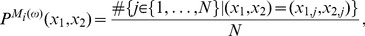
(13)where 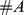 denotes the cardinality of the set 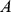. In order to obtain a distribution over test statistics for the reference distribution under review, the divergence 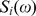 between the original reference distribution and each of the empirical distributions of the samples 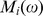 is calculated. This distribution is compared to the divergence 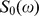 between the reference distribution and the empirical distribution of the observed data. The location of 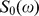 within the empirical distribution of test statistics 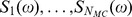 yields a *p*-value:
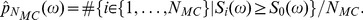
(14)


The spike count distribution is a discrete distribution and, therefore, the distribution of test statistics is also discrete. The formalism requires a properly randomized distribution to be rigorous [33]. Hence, we need to take into account ties of the test statistics. To accomplish this a simple procedure called tie breaking can be applied [33]. Essentially, the procedure orders test statistics randomly whenever they are the same.

Formally, for each test statistic 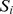 an i.i.d. random variable 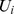 is drawn from the uniform distribution on the interval 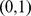. The test statistics are then reordered according to 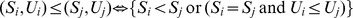.

The *p*-value has to be corrected for the finite number 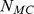 of Monte Carlo samples and is given by [33]:
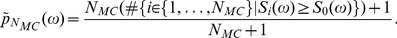
(15)


The *p*-value is then maximized over the nuisance parameters (i.e., the correlation coefficient or parameters of the marginals) that span the space of maximum entropy distributions 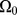:
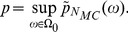
(16)The procedure results in a test in which the false rejection rate is guaranteed to be below the 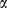-level [33], thus allowing us to examine evidence for higher-order correlations that is reflected in the particular divergence measure of interest. Note that the power of the test slightly increases with the number of Monte Carlo samples. However, the power is primarily affected by the sample size (cf. Section “Comparison to Likelihood Ratio Test”).

In this paper we consider two divergence measures that are based on common information measures: The entropy difference:
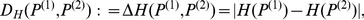
(17)and the mutual information difference:
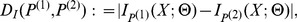
(18)where 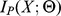 is given by
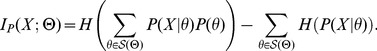
(19)

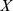
 and 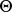 are random variables with conditional distributions 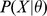 of a given realization 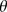 from 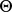.

If the test rejects the second-order hypothesis then we can conclude that significant differences exist in terms of the information measure between the data and the predictions of models neglecting higher-order correlations. For the mutual information between stimuli and neural responses, for example, this means that the mutual information estimates obtained for data and models are significantly different. Hence our approach tests for higher-order correlations which are relevant for a particular analysis task. Consider the extreme example that only the probability of both neurons being silent (no spikes of both neurons) is of interest. Then one could apply a divergence measure which takes only the probability of that spike count pair into account. In contrast, other common approaches would just quantify a general divergence of the distributions which might not be of interest at all.

Additional divergence measures are discussed in the supporting information (cf. Section “Alternative Divergence Measures” in Text S1).

### Recording Procedures

The experimental procedures have been described previously [34]. Here, we briefly repeat the description for completeness. The experiment was performed under protocols approved by MIT's Animal Care and Use Committee. The animal was anesthetized and paralyzed. Neural responses were measured to drifting high-contrast square-wave gratings of 16 directions. Each drifting grating had a frequency of 1 Hz. In the control conditions the 16 drifting gratings were presented for 10 trials each for a total of 160 trials, 2.5 s each presentation. We selected the first 7 trials each to match the number of trials of the second condition. In the adaptation condition one grating of fixed orientation moving randomly in two opposite directions was presented for a duration of 2 min. Afterward, the drifting test gratings with 16 different directions were presented randomly for 2.5 s each (7 trials per grating), preceded by a 5 s “topping-up” presentation of the adapting orientation. Multiple simultaneous extra-cellular recordings were made using tungsten microelectrodes at cortical depths between 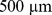 and 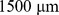 in the primary visual cortex. Responses from 11 cells were recorded whose orientation preferences in the control condition covered the entire orientation range. The signal was amplified and thresholded to obtain spike trains.

### Data Analysis

The spike trains were binned into non-overlapping time intervals and the number of spikes was counted within these intervals. The length of the intervals was varied between 10 ms and 400 ms. Three recurrences of the same grating appeared in each of the 2.5 s presentations. The sample pool of each model and each test was based on the orientation of the grating stimulus (trials with opposing directions of movement were combined), on the time points within an iteration of the drifting grating and on the two experimental conditions. This yielded a total of 42 repetitions each for the control and adaptation conditions, the 8 orientations and for each of the time intervals with stimulus presentation. Samples from within an iteration were not mixed to prevent confounding effects from varying rates. Therefore, separate tests were used for every time step and every neuron pair. The false discovery rate of the multiple testing rejections was corrected using the Benjamini-Hochberg procedure [35] with a significance level 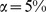. This multiple testing procedure controls the expected proportion of falsely rejected hypotheses in a multiple inference setting. It was applied over all pairs, all time bins for a given bin size and, in the case of entropy difference as the divergence measure, over all stimulus orientations. The correction was not applied over all bin sizes, because the potential absence of higher-order correlations is treated as a separate hypothesis for every bin size.

## Results

### Validation of the Test on Artificial Data

We devised a test that determines whether the dependence structure of spike count distributions is sufficiently well characterized by the second-order statistics (cf. “Materials and Methods”). The test was applied to spike count samples drawn from three different families of bivariate distributions: (1) a family of maximum entropy distributions constrained by rates and linear correlations only (ME), (2) a family of distributions with higher-order correlations but vanishing linear correlations (M1), and (3) a family of distributions with higher-order correlations in the presence of limited linear correlations (M2). Marginals 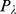 were always Poisson distributed, i.e.
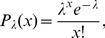
(20)where 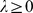 is the mean and 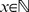 is the spike count variable.

**Figure 2:**
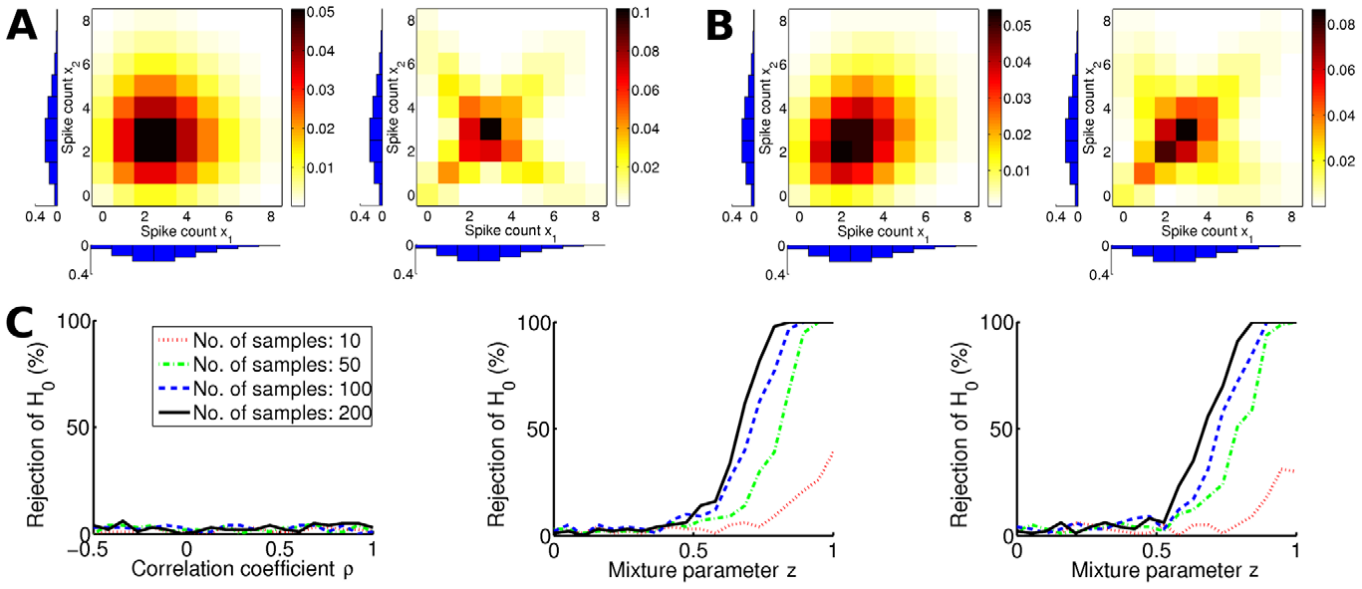
Evaluation of the maximum entropy test on artificial data. (A) Probability mass functions of the maximum entropy distribution 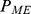 (left) and the Gaussian copula based distribution 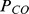 (right) of the mixture distribution 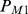 with a linear correlation coefficient of 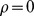. The Poisson marginals (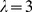) are plotted along the axes. (B) Same as A but for the mixture distribution 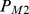 with a linear correlation coefficient of 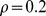. (C) Percent rejections of the null hypothesis using the entropy difference as the divergence measure. Significance level was 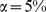. Rejection rates were estimated over 100 tests. Different lines correspond to different numbers of samples drawn from the candidate distribution: 10 (red dotted line), 50 (green dash-dotted line), 100 (blue dashed line), and 200 (black solid line). (Left) Results for the 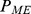 family for varying correlation coefficient 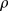. (Center) Results for distributions from the 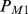 family (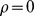) for varying mixture parameter 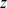 (cf. Figure 2 A). (Right) Same for 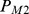 (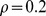, cf. Figure 2 B)). Poisson rate was 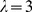 for all candidate distributions (corresponding to 30 Hz and 100 ms bins). Simulated annealing [45] was applied to maximize the *p*-value (cf. Text S1). Number 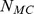 of Monte Carlo samples was 1000.

The maximum entropy distributions ME served as reference distributions and were constructed according to Equations 6–9 and varying correlation coefficient. In order to investigate the power of the test we constructed two distribution families M1 and M2, which included higher-order correlations. These families are based on so-called copulas, which allow us to construct multivariate distributions with Poisson marginals and higher-order correlations [23], [36]. The families M1 and M2 consisted of two components:
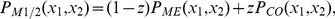
(21)where 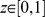 is a mixture parameter, 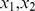 are spike counts. The two components were defined as a maximum entropy distribution of the family ME (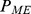, cf. Equations 6–9 with Poisson marginals as in Equation 20 and Figure 2 A, left, and 2 B, left) and a copula-based distribution 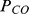 (cf. Figure 2 A, right, for M1 and 2 B, right, for M2) which was a mixture distribution by itself and which showed significant higher-order correlations [23]:
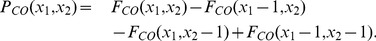
(22)The cumulative distribution function (CDF) 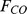 is defined as
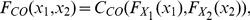
(23)where the CDF's of the Poisson marginals are defined as
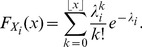
(24)

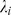
 is the rate parameter of element 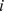. Equations 22 and 23 hold for every copula-based distribution with discrete marginals. Linear and higher-order correlations are specified by the applied copula. The copula 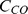 of the model is defined as a Gaussian mixture copula
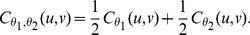
(25)The bivariate Gaussian copula family is defined as
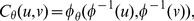
(26)where 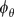 is the CDF of the bivariate zero-mean unit-variance normal distribution with correlation coefficient 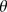 and 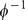 is the inverse of the CDF of the univariate zero-mean unit-variance Gaussian distribution.

We chose the Gaussian copula model to construct 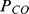, because it can be used to systematically introduce higher-order correlations while keeping the marginal distributions unchanged. The marginal Poisson distributions have one rate parameter each whereas the Gaussian copula family has one parameter which affects the linear correlation coefficient and higher-order correlations of the bivariate model, but not the marginal distributions. The resulting linear correlation coefficients can be positive or negative. We can therefore apply a mixture of two Gaussian copulas with two opposite correlation coefficients. This yields a model with an overall linear correlation coefficient that is arbitrarily small. The higher-order correlations, on the other hand, can still be strong. These dependencies are visible as a cross in the probability mass function (Figure 2 A, right).

In order to generate strong higher-order correlations we set 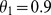 and numerically adjusted 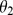 for each 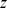 to obtain a certain linear correlation coefficient for 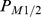 (correlation coefficient 0 for model M1 and 0.2 for model M2).

For family M1 the correlation coefficient of both 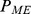 and 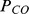 was set to 0 (no linear correlation) while for family M2 the correlation coefficient was set to 0.2 (weak positive linear correlation). Therefore, the linear correlation coefficient (and the marginals) of families (M1) and (M2) were by construction independent of the mixture parameter 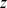. The mixture parameter 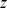, however, controlled the strength of the higher-order correlations. For 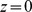, 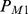 and 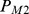 corresponded to maximum entropy distributions with linear correlations only, while 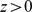 led to distributions 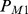 and 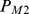 with higher-order correlations. A mixture distribution can be interpreted as a model with multiple common inputs which are active at different times. In the 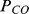 model there were two mixture elements with opposite correlation coefficients corresponding to inputs that produce correlated or anticorrelated responses. For 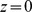 these inputs were absent. Increasing 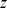 corresponded to increasing the strengths of these inputs.

Figure 2 C shows the results of the maximum entropy test for higher-order correlations for several members of the M1 and M2 families of bivariate spike count distributions for the entropy difference (Equation 17) as the divergence measure. All subfigures show the percent rejections of the null hypothesis 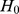 (cf. previous section) on a significance level 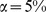, i.e. the rejections of the hypothesis that higher-order correlations did not significantly influence the estimated values for the entropy of the distributions M1 and M2. The rejection rates were estimated over 100 trials. Different lines represent different sample sizes (10, 50, 100 and 200) to which the test was applied.

Figure 2 C (left) shows the percent rejections of 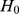 for data samples from maximum entropy distributions 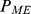 with different linear, but no higher-order, correlations present. As expected, the achieved Type I error (i.e. rejections despite absence of higher-order correlations in the underlying distribution) was small and the acceptance rate of 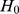 was close to the desired value of 95%. The center and left subfigures in Figure 2 C show the percent rejections of 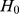 for different strengths of higher-order correlations of samples drawn from the M1- (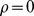) and M2-distributions (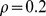). The larger the mixture parameter 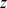 the higher the percent rejection of 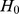, i.e. percent rejections increases for increasing strength of the higher-order correlations. Moreover, Type II errors (no rejections despite presence of higher-order correlations in the underlying distribution) decrease for increasing sample sizes. Therefore, the test can successfully detect moderately strong higher-order correlations in artificial data even when the sample size is on the order of 50. Since the results for the M1- (Figure 2 C, center) and M2-distributions (Figure 2 C, right) were similar, the test was insensitive to the presence of linear correlations. Additional results for a Poisson rate 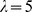 (corresponding to 50 Hz and 100 ms bins) are shown in Figure S2 in Text S1 and resemble those of Figure 2 C.

#### Comparison to likelihood ratio test

We compared the proposed Monte Carlo maximum entropy test to a standard approach for statistical testing: the likelihood ratio test [37]. This test evaluates whether a reduced model provides a fit that is as good as a full model. In this case, the full model is the multinomial distribution and the reduced model is the maximum entropy distribution. Parameters are estimated by maximizing the likelihood of the respective model. Let 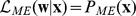 denote the likelihood of the maximum entropy distribution and 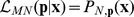 the likelihood function of the multinomial distribution (cf. Equations 6 and 10). Then the likelihood ratio statistic is given by
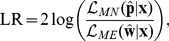
(27)where 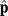 and 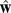 are the maximum likelihood estimators of the distribution parameters. For sufficiently large sample size, the LR test statistic is 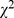 distributed with 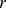 degrees of freedom, where 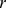 denotes the number of additional free parameters in the full model compared to the maximum entropy model. Note that for limited sample sizes the 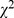 distribution is not necessarily exact.

**Figure 3:**
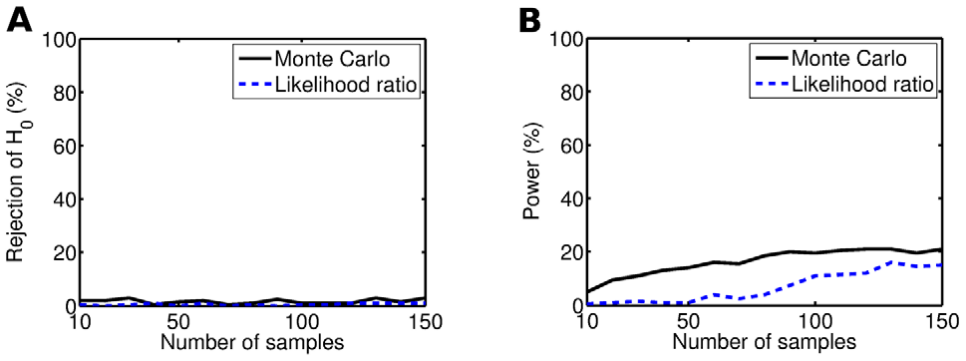
Effect of sample size on the Monte Carlo maximum entropy test results (solid black line) and on the maximum likelihood ratio test results (dashed blue line) with Poisson rate 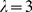. The entropy difference was used as a divergence measure. Significance level was 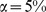. Rates were estimated over 100 trials. ( A) Percent rejections of the maximum entropy hypothesis. Data were sampled from maximum entropy distributions with random correlation strengths. ( B) Percent rejections of the null hypothesis. Data were sampled from a copula-based mixture model with uniformly random mixture parameter (cf. 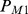, Equation 21).

We compared the likelihood ratio test to the proposed Monte Carlo test for varying sample sizes. Figure 3 A shows the percentages of rejections of the maximum entropy hypothesis for data that were sampled from a maximum entropy model (Type I error). For both tests the percentages do not depend on the number of samples. The percentages of rejections are generally smaller for the likelihood ratio test. Both tests, however, have a rejection rate below the significance level 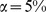.

We also evaluated the power of the tests as a function of sample size. We expected that the Monte Carlo test would perform better for small sample sizes, because the 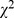 distribution is less accurate in these cases. Figure 3 B shows the power with respect to the copula mixture models (cf. Section “Validation of the Test on Artificial Data”) with a mixture parameter that was sampled uniformly. The figure shows that the power of the proposed test is indeed much greater when the sample size is small. The likelihood ratio test has almost no rejections for sample sizes below 50, whereas the Monte Carlo test rejects between 5% and 20% of the maximum entropy hypotheses. But also for medium sample sizes the power of the Monte Carlo test surpasses that of the likelihood ratio test.

#### Impact of autocorrelations

Neural spike trains typically have autocorrelation structure which is known to affect estimates of correlations [38]. We explored the impact of autocorrelations on the proposed Monte Carlo maximum entropy test by simulating gamma processes with varying refractory periods. Interspike intervals were sampled from the gamma distribution
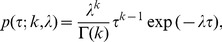
(28)where 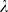 is the rate of the process (set to 30 Hz) and 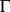 is the gamma function. The exponential distribution is a special case of the gamma distribution when 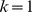. In this case we obtain a Poisson process, whereas for 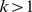 the gamma process has a refractory period. We varied 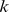 between 1 and 2. The processes become more regular when we increase 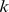. Autocorrelations therefore increase with increasing 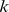.

**Figure 4:**
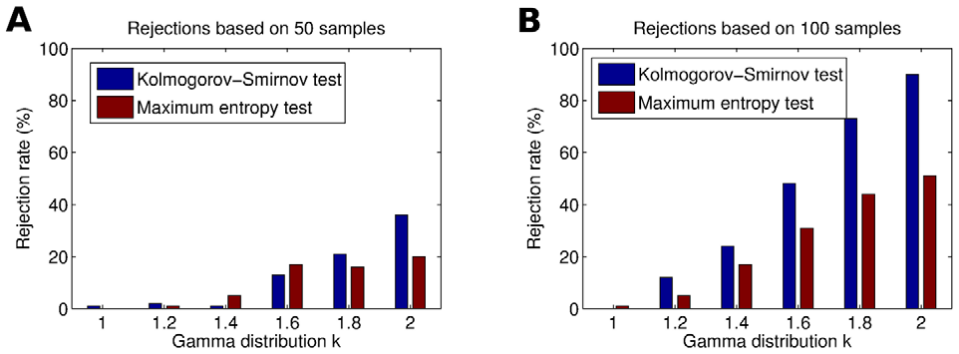
Effect of autocorrelations on the Monte Carlo maximum entropy test results (blue) and on the discrete Kolmogorov-Smirnov test results (red). Interspike intervals of two concurrent spike trains were sampled from a gamma distribution with constant rate 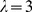 and gamma parameter 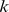. Spike counts were calculated over subsequent 100 ms bins. The entropy difference was used as a divergence measure. Significance level was 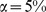. Rates were estimated over 100 trials. ( A) 50 spike count pairs were sampled for each test trial. ( B) 100 spike count pairs were sampled for each test trial.

In each trial we simulated two concurrent spike trains. The goodness-of-fit of a Poisson process was assessed with a Kolmogorov-Smirnov test at a 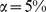 significance level (c.f. Section “Poisson Goodness-of-fit Tests” in Text S1). Moreover, we binned the spike trains into 100 ms intervals and calculated simultaneous spike count pairs. We then applied our proposed maximum entropy test to the spike count pairs. Figure 4 shows the rejections rates for ( A) 50 samples (corresponding to spike trains of length 5 s) and ( B) 100 samples (corresponding to spike trains of length 10 s) over 100 trials. The rejection rates of both tests increase with 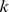. This reflects that the deviation from Poisson processes increases. Furthermore, both tests have greater rejection rates when applied to 100 samples ( B) than when applied to 50 samples ( A).

In almost every case, rejection rates of the maximum entropy test are lower compared to the Kolmogorov-Smirnov test. This shows that the Kolmogorov-Smirnov test is more sensitive to autocorrelations of gamma processes and has more statistical power than our proposed maximum entropy test. This comes as no surprise, since the Kolmogorov-Smirnov test operates directly on the spike trains whereas the maximum entropy test assesses the spike counts without any information of spike timing.

### Application to Data Recorded from Cat V1

**Figure 5:**
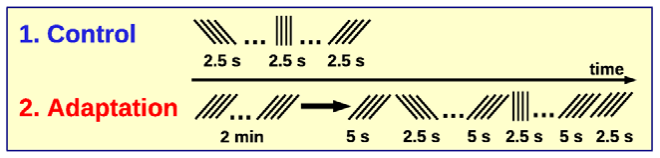
Illustration of the control and adaptation protocols.

The new maximum entropy test was applied to neural spike trains recorded from the primary visual cortex of anesthetized cat during visual stimulation [34]. The protocols of the neurophysiological experiments are depicted in Figure 5. Drifting gratings of random orientations between 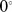 and 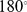 (resolution 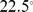) were presented during two conditions. In the control condition, each (test) orientation was presented for 2.5 s. In the adaptation condition, an initial block (2 min) of one grating of fixed orientation was followed by random presentations of the 8 orientations (2.5 s). Each of these (test) gratings was preceded by a 5 s presentation of the adapted grating in order to maintain the orientation effects. Simultaneous neural activity from 11 cells was recorded by multiple electrodes in V1. The resulting spike trains were binned and transformed to spike count sequences. We thereby obtained a total of 42 repetitions for each condition, orientation and non-overlapping spike train bin of varying length.

Application of the maximum entropy test requires a maximization of the *p*-values over the nuisance parameters (Equation 16) which include the marginal distributions. Because a maximization over all possible marginal distributions would have been unfeasible, we made a parametric assumption and described all marginal distributions by Poisson distributions (Equation 20) with rate parameters 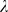 as the only nuisance parameters. Note that parameter values differed between neurons, conditions, orientations and spike train bins.

The stimulus grating was drifting with a frequency of 1 Hz. The size of the spike count bins was varied between 10 ms and 400 ms. The changing stimulus-driven rate might therefore violate the Poisson assumption depending on the size of the bin. Several statistical tests were applied to check whether our assumption should be rejected (cf. Section “Poisson Goodness-of-fit Tests” in Text S1). Indeed, neither a single neuron Monte Carlo goodness-of-fit test for Poisson statistics nor a multivariate Monte Carlo goodness-of-fit test for the product distribution (after removing all dependencies) led to rejections of the Poisson hypothesis for any of the bin sizes. Taken together, these findings do not provide any evidence against our assumption of Poisson-distributed marginals.

Furthermore, we applied Kolmogorov-Smirnov tests based on the discrete time rescaling theorem [39] quantifying the interspike interval statistics (cf. “Poisson Goodness-of-fit Tests” in Text S1). Although a discrete Poisson distribution of the spike counts does not necessarily imply interspike interval statistics that follow a Poisson spike generating process, the reverse always holds. For rates estimated in 100 ms bins, the rejection rates of the Poisson process hypothesis were below 5%. For greater bin sizes (200 ms, 400 ms), the rejection rates increased. For a detailed discussion, see “Poisson Goodness-of-fit Tests” in Text S1.

**Figure 6:**
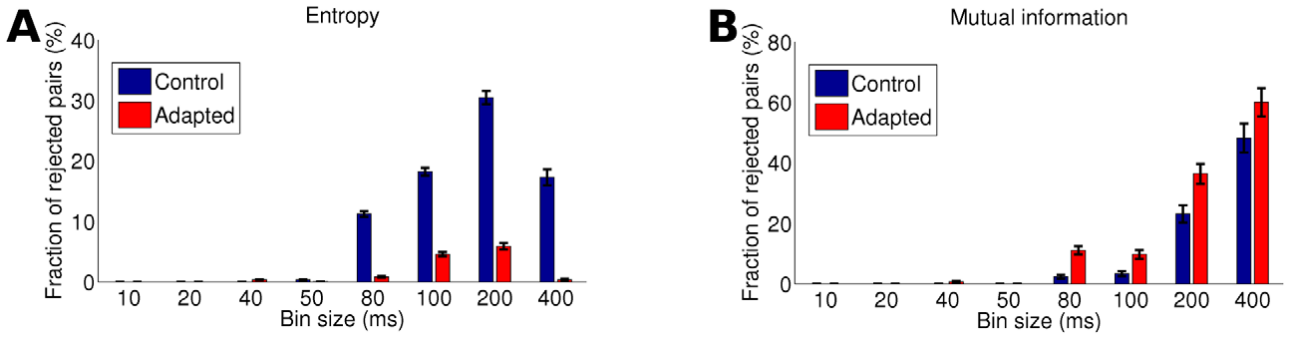
Results of the maximum entropy test for data recorded from area V1 of anesthetized cat. The evaluation was performed separately for the control and adaptation conditions. ( A) Fraction of neuron pairs rejected by the Monte Carlo maximum entropy test with the entropy difference as the divergence measure (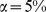) and for different bin sizes. ( B) Same as in A but using the mutual information difference. Rejection rates were averaged over all neuron pairs and all time bins. Simulated annealing [45] was applied to maximize the *p*-value (cf. Text S1). Number 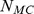 of Monte Carlo samples was 1000. The false discovery rate of the rejections was corrected using the Benjamini-Hochberg procedure [35].

We applied separate maximum entropy tests to all 55 neuronal pairs and to all time bins (non-overlapping time intervals locked to the start of a grating presentation at varying latencies, cf. Section “Data Analysis”). Figure 6 A shows the results for the entropy difference (Equation 17) as the divergence measure. The rejections were corrected for multiple inferences and averaged over neuron pairs, stimuli and time bins for given bin sizes (cf. Section “Data Analysis”). The fraction of rejected pairs increased with increasing bin size until it reached a maximum at 200 ms. Therefore, as bin size increases, more and more neuron pairs show significant differences between the entropy estimated directly from the data and the entropy estimated using models which neglect higher-order correlations.

The smaller value for a bin size of 400 ms could already be a consequence of the central limit theorem: For finite rates and in the limit of large bin sizes the distribution of the spike counts converges to a bivariate normal distribution which is an instance of a second-order maximum entropy distribution.

Figure 6 B shows the results for the difference in mutual information (Equation 18) as the divergence measure. The mutual information is calculated between the orientations of the test-gratings and the corresponding spike counts: 
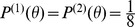
 were the flat distributions over gratings, 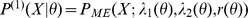 was the maximum entropy reference distribution subject to Poisson rates 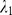 and 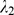 and a correlation coefficient 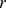 for each of the eight stimulus values. 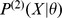 was the empirical distribution over the data set or Monte Carlo sample.

The fraction of rejected pairs increased with bin size showing that for many bin sizes there was significant evidence for higher-order correlations that was reflected in the mutual information for a substantial number of pairs. However, contrary to the results for the entropy difference, the number of rejections was significantly higher in the adapted condition than in the control condition for bin sizes 80 ms, 100 ms and 200 ms (paired *t*-test, 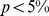). For the adaptation condition, there was much more evidence for higher-order correlations that was reflected in the mutual information than in the entropy. This indicates that for many bin sizes divergences from the maximum entropy distribution were more stimulus specific after adaptation even though they were smaller.

For the entropy difference as the divergence measure, rejection rates do not vary with stimulus orientation. For both the entropy difference and the mutual information difference, the data suggest that rejection rates tend to increase towards the end of the trial (data not shown).

**Figure 7:**
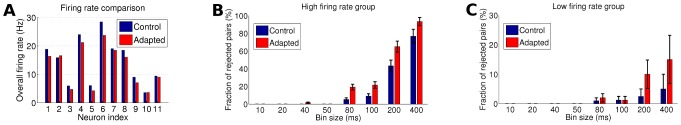
Subpopulation analysis of the data that are presented in Figure 6 C. (A) Overall firing rates of the 11 neurons in the data set from Figure 6 for the control and adaptation conditions. The rates were averaged over all stimuli. ( B) Fraction of neuronal pairs rejected by the maximum entropy test with the mutual information difference as the divergence measure (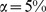) for the high firing rate (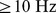, cf. A) population of neurons. ( C) Same as in B but for the low firing rate population (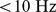). Rejection rates were averaged over all neuron pairs and all time bins. Simulated annealing [45] was applied to maximize the *p*-value (cf. Text S1). Number 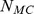 of Monte Carlo samples was 1000. The false discovery rate of the rejections was corrected using the Benjamini-Hochberg procedure [35].

Figure 7 A shows the overall firing rates of the individual neurons in the control and the adaptation conditions. The data suggest the existence of a high firing rate (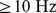) and a low firing rate (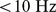) population. Figures 7 B, C show the results of the maximum entropy test for the difference in the mutual information as a divergence measure separately for both populations. The figures show that the rejected pairs were significantly higher for the high firing rate population for bin sizes 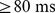 in both the control and the adaptation condition (paired *t*-test, 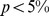). Moreover, the rejection rates in this subpopulation were significantly higher in the adaptation condition than in the control condition for these bin sizes (paired *t*-test, 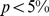), which did not hold for the low firing rate population.

We explicitly estimated the mutual information between the bivariate spike counts of neuronal pairs and the stimulus set using (1) the best fitting second-order maximum entropy distribution, which neglects higher-order correlations and (2) a non-parametric method involving a bias correction for small sample sizes, where higher-order correlations are included in principle (cf. Section “Subpopulation Structure of Recorded Neurons” in Text S1). The relation between these estimates can roughly illustrate the order of impact of higher-order correlations even though the number of samples is insufficient to obtain unbiased mutual information estimates. Results show that when higher-order correlations are taken into account a bimodal distribution of mutual information values emerges, with modes coinciding with the low firing rate (for small mutual information values) and high firing rate (for high mutual information values) population (cf. Figure S5 in Text S1).

## Discussion

We devised a maximum entropy test that assesses higher-order correlations in terms of an information theoretic analysis. The biggest advantage of the method is the small number of samples that is required. We demonstrate that the test can be useful even when the number of samples is on the order of 50. Our approach has the advantage of being able to test for higher-order correlations, which are relevant for a particular analysis task, rather than in terms of a general divergence measure, which might not be of interest at all.

A divergence measure that is based on mutual information can be applied to quantify evidence for higher-order correlations that is reflected in mutual information. Suppose two stimuli 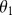 and 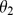 are present with conditional bivariate spike count distributions 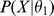 and 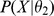. Let 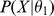 follow a maximum entropy distribution and 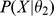 follow the mixture distribution of Section “Validation of the Test on Artificial Data” with the same correlation coefficient. Then the mutual information is 0 for 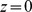 and increases with 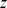. The mutual information difference can therefore quantify divergence in terms of a measure of interest.

The test that we presented is restricted to neuronal pairs. Therefore, multivariate higher-order correlations that are not detectable in bivariate distributions would be overseen. The maximum entropy hypothesis would be accepted in spite of such correlations being present. The number of rejections, however, could only increase but not decrease. In principle, a theoretical generalization of the test to an arbitrary number of neurons is straightforward. Practical computation time and memory requirements, however, increase exponentially with the number of neurons. It is not clear whether higher-order correlations that are not detectable in bivariate distributions are particularly important for neural systems. From a generative viewpoint, however, this condition would impose strong constraints on the statistics of the neural responses: on the order of 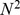 constraints would be necessary for a population of 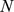 neurons to keep the neurons linearly uncorrelated while higher-order correlations would need to be stimulus specific.

If the number of samples is on the order of 50 and higher-order correlations are present then mutual information estimations are unreliable. The test with the mutual information difference as the divergence measure includes the calculation of mutual information values. This is acceptable for several reasons: (1) The goal of the method is not to yield an estimate of the mutual information, but rather to quantify the divergence of the data compared to the reference family of distributions. (2) Simple bias correction techniques that depend on the number of samples (e.g. the Miller-Madow bias corrections) are implicitly present in the test because the divergence is the difference of the mutual information and therefore, any additive biases vanish. (3) The test searches in the parameter space of maximum entropy distributions and calculates their mutual information values. Under the null hypothesis this means that the true mutual information is among those that we consider. Since we use the worst case *p*-value, the test is reliable even if the number of samples is insufficient to estimate mutual information.

We cannot make sure that the spike counts are actually Poisson distributed even though we applied several statistical tests to ascertain that this assumption is not unreasonable. We explored the impact of deviations from the Poisson distribution by applying the maximum entropy test to gamma processes. Naturally, the test rejects the maximum entropy distribution with Poisson marginals if the deviations of the gamma process from the Poisson process are too strong. However, we also compared the rejection rates of the maximum entropy test to the rejection rates of the Kolmogorov-Smirnov test. The Kolmogorov-Smirnov test turned out to be more sensitive to these deviations than the proposed maximum entropy test. This suggests that the V1 rejection rates of the Kolmogorov-Smirnov test should be much greater if the Poisson assumption was the reason for the strong rejection rates of the maximum entropy test. In general, one could assume more flexible marginals if the Poisson hypothesis must be rejected. We propose the negative binomial and the binomial distribution as alternatives in the supporting information (cf. Section “Alternative Marginal Distributions” in Text S1).

It might come as a surprise that the application of the Poisson goodness-of-fit tests to the V1 data yielded so little Poisson rejections, given that spike trains are non-stationary and typically have strong autocorrelations. We emphasize that we applied separate tests for every bin: spike counts from subsequent bins of a single trial were not modeled by a single distribution but by separate distributions. We make no assumptions about the relation of these subsequent models. The test, therefore, cannot detect any higher-order correlations that have a time lag beyond the length of the bin. Moreover, we assume that spike counts of a given bin can be described by a single stationary distribution across trials. In principle, additional tests could be applied to bin pairs with a fixed lag in order to detect higher-order correlations that have a time lag beyond the length of the bin.

Spike counts are calculated within subsequent bins of a given length. It is well known that higher-order correlations are of no relevance if the bin size is very small. In this case the marginals are essentially binary: either there was a spike present in the bin or not. The bivariate distribution table of these spike counts is characterized by only three probabilities (the table has four values but the last one is fixed by the constraint that the probabilities sum to one). Thus the correlation coefficient is sufficient to characterize the dependency structure for very small bin sizes. For larger bin sizes higher-order correlations can be important, however, we assume that the bin size is already fixed before the test is applied. The goal of our analysis is not to find a parameter regime where higher-order correlations are necessary. Instead, there was a separate hypothesis inference for each bin size. As such, we applied a multiple inference procedure to analyze the data that were recorded from V1. Multiple inference procedures do not need a multiple testing correction [40]. Instead, we applied a multiple testing correction over all pairs and all time bins for a given bin size but not over all bin sizes. If the goal of the analysis would have been to identify a particular bin size for which higher-order correlations do matter or if the test is applied in an exploratory study over multiple bin sizes to determine whether more data should be collected, then the multiple testing correction should as well be applied over all bin sizes.

Previously, it was shown that orientation adaptation in cat V1 neurons results in shifts of the preferred orientation [34] and in changes of the distribution of linear correlations between neuronal pairs [1]. In our “proof of principle” example we investigated whether higher-order correlations change and whether they have a significant impact on information theoretic quantities. Taken together, the results of our analysis provide evidence for condition-dependent influences of higher-order correlations on the estimation of entropy and mutual information for many spike count bin sizes. Furthermore, our analysis suggests the existence of different subpopulations of neurons with a different higher-order correlation structure.

The purpose of the test is to show whether higher-order correlations must be taken into account when a particular kind of analysis is planned for an experimental data set and not to provide in depth insight into the structure of these associations. The advantage of the test lies in the small numbers of samples it needs to detect the presence of analysis-relevant higher-order correlations, hence it can be applied to a smaller exploratory study. If the maximum entropy hypothesis is rejected, one learns two things: (1) One should not perform the planned analysis on the given data and (2) one should redo the experiment and increase the number of data, such that higher-order associations can be reliably estimated. In the supporting information, we briefly describe generalized linear models (GLMs) as one particular option for modeling higher-order associations (cf. Section “Modeling Higher-order Correlations” in Text S1). For a more detailed description of GLMs we refer the reader to previous studies [41]–[44].

The new test provides a convenient way to investigate the sufficiency of second-order dependency models, and is especially useful when the number of samples per condition is small - a typical situation in electrophysiology. The application of the test to data recorded in primary visual cortex provides a proof of principle for the usefulness of our method.

## Supporting Information

Text S1 Supporting text providing a detailed description of the optimization procedure, of Poisson goodness-of-fit tests, of alternative marginal distributions and divergence measures. It also provides a discussion of modeling higher-order correlations and of the subpopulation structure of the recorded neurons.
(PDF)
