# Supplementary material for: A Maximum Entropy Test for Evaluating Higher-Order Correlations in Spike Counts
Source: PLoS Comput Biol. 2012 Jun 7;8(6):e1002539. doi: 10.1371/journal.pcbi.1002539 (PMC3369943; doi:10.1371/journal.pcbi.1002539)
Supplement: Text S1 — Supporting text providing a detailed description of the optimization procedure, of Poisson goodness-of-fit tests, of alternative marginal distributions and divergence measures. It also provides a discussion of modeling higher-order correlations and of the subpopulation structure of the recorded neurons. (PDF) [file pcbi.1002539.s001.pdf]

Text S1

1 Optimization of the Nuisance Parameters

The nuisance parameters (rates of the marginals and correlation coefficients) of the distributions are unknown. We can just obtain estimates of these parameters based on the available data set. However, our goal is to construct a test which is applicable even when the number of samples is very small. In this case, the estimates of the nuisance parameters are generally unreliable. Instead of relying on specific estimates of the nuisance parameters, we maximize the p-value over all possible nuisance parameters. The cost function of the optimization is given by the negative p-value that is obtained for a specific set of nuisance parameters of the distributions.

In order to compute the entropy and the mutual information of a particular distribution we restricted the support of the spike counts to the set $\{0, 1, \ldots, b\}$, where b was selected such that the residual mass of the distribution was less than 0.1% of the total mass:

$$b = \max\left\{\hat{b} \in \mathbb{N} : 1 - \sum_{i,j=0}^{\hat{b}} P(x_1 = i, x_2 = j) < 0.001\right\}.$$

The sets of possible nuisance parameters are continuous. There are many algorithms for optimizing a nonlinear function over continuous data sets. Grid search [1] is one option for optimizing the nuisance parameters globally. However, when the mutual information is used for the divergence measure three nuisance parameters have to be considered for every conditional probability distribution $P(X_1, X_2|\theta)$, i.e. for every stimulus θ of the stimulus set. This made it unfeasible to maximize the p-value using grid search when the mutual information is used as the divergence measure, since the number of parameter combinations is too big. Therefore, we applied simulated annealing [2] to maximize the p-value in all of the analyzes of the main article. For the initial values of the simulated annealing optimization we chose the sample means for the Poisson rate parameters and the sample correlation coefficients for the correlations of the maximum entropy distributions.

We used the default `simulannealbnd` implementation from the MATLAB R2011b Global Optimization Toolbox. The initial temperature T_{init} was set to 100. The temperature was updated according to $T(k) = 0.95^k T_{init}$ with k the iteration number. These were default values of the toolbox. New points

were generated with uniform random directions and steps of length $T(k)$. If the new objective function value was less than the old, the new point was always accepted. Otherwise, the new point was accepted with probability

$$\frac{1}{1+\exp\left(\frac{\Delta}{T(k)}\right)},$$

where Δ is the difference in objective function values. Rates were bounded by zero (lower bound) and two times the maximum sample rate (upper bound) and correlation coefficients were bounded by -1 (lower bound) and 1 (upper bound). Points outside of the boundary region were never accepted. The algorithm terminated after the average change in value of the objective function in 10 iterations was less than 10^{-6}.

Maximization problems are harder to solve if many local maxima are present, because simulated annealing can converge to a local maximum instead of the global maximum. This is likely to happen if the global maximum is peaked with small support. In order to make sure that simulated annealing is appropriate for this maximization task we plotted p-values as a function of the nuisance parameters for the entropy difference as the divergence measure and for input data sampled from a maximum entropy distribution showing an acceptance (Figure S1A, B) and from a higher-order mixture distribution showing a rejection (Figure S1C, D). The p-values are symmetric with respect to the rate parameters of the Poisson marginals. In both cases, the plateau areas are localized in a relatively small region of the parameter space with the empirical parameter estimates being close to the maxima.

Note that the true distribution does not necessarily have the highest p-value, because a low value reflects strong divergence while the highest value does not necessarily reflect the greatest similarity. The landscape also depends on the data sample that the p-values are based on. For very low and high rates the distributions are very dissimilar in terms of the entropy difference.

2 Test Validation for Different Rates

Figure 2C of the main article shows the rejection rates of the maximum entropy test applied to the data from the mixture model $P_{M1/2}$. A similar test application is shown in Figure S2. The only difference are the rates of the Poisson marginals: the rates are set to $\lambda = 5$ (corresponding to 50 Hz for 100 ms bins). The results are resembling those of Figure 2C and thus demonstrate that the maximum entropy test successfully detects higher-order correlations for different rates.

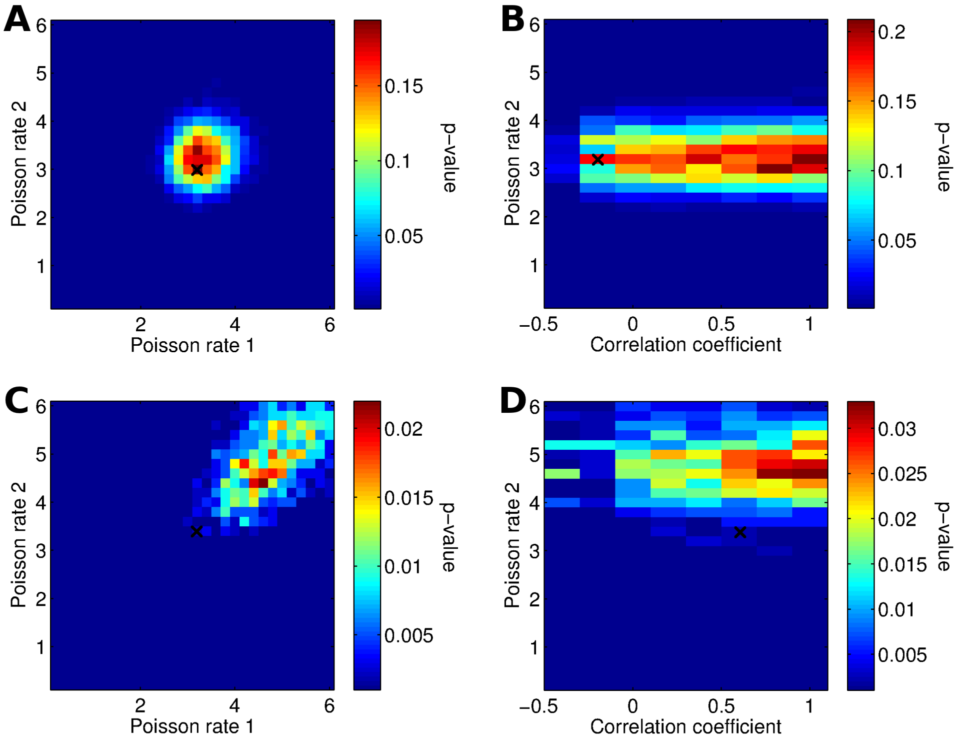

Figure S1. Optimization landscapes of the nuisance rate parameters θ_1 and θ_2 and the nuisance correlation coefficient θ_3 (resolution 0.2). Number N_{MC} of Monte Carlo samples was 1000. Number of input samples was 50. Empirical parameter estimates are marked by crosses.
(A, B) Input data were sampled from a maximum entropy model with Poisson marginals ($\lambda = 3$) and a correlation coefficient $\rho = 0.2$. H_0 is not rejected at $\alpha = 5\%$, because p-values exceeding 5% occurred.
(C, D) Input data were sampled from model P_{M1} (cf. Equation 21) with Poisson marginals ($\lambda = 3$). H_0 is rejected at $\alpha = 5\%$, because no p-value exceeds 5%. (A) The p-value as a function of the nuisance rate parameters θ_1 and θ_2 for nuisance correlation coefficient $\rho = 0.2$. (B) The p-value as a function of the nuisance correlation coefficient θ_3 and the nuisance rate parameter θ_2 for $\theta_1 = 3$. (C) The p-value as a function of the nuisance rate parameters θ_1 and θ_2 for nuisance correlation coefficient $\rho = 0$. (D) The p-value as a function of the nuisance correlation coefficient θ_3 and the nuisance rate parameter θ_2 for $\theta_1 = 4.5$.

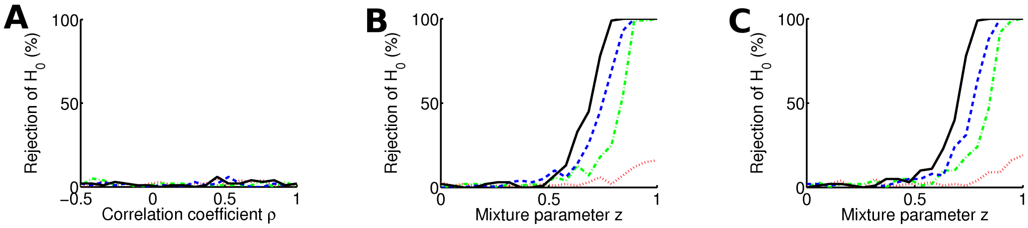

Figure S2. Percent rejections of the null hypothesis with Poisson rate $\lambda = 5$ for all candidate distributions (corresponding to 50 Hz and 100 ms bins). The entropy difference was used as the divergence measure. Significance level was $\alpha = 5\%$. Rejection rates were estimated over 100 trials. Different lines correspond to different numbers of samples drawn from the candidate distribution: 10 (red dotted line), 50 (green dash-dotted line), 100 (blue dashed line), and 200 (black solid line). (A) Results for the P_{ME} family for varying correlation coefficient ρ. (B) Results for distributions from the P_{M1} family ($\rho = 0$) for varying mixture parameter z (cf. Figure 2A). (C) Same for P_{M2} ($\rho = 0.2$, cf. Figure 2B)). Simulated annealing was applied to maximize the p-value. Number N_{MC} of Monte Carlo samples was 1000.

3 Poisson Goodness-of-fit Tests

We applied three Poisson goodness-of-fit tests to check whether there is evidence against the Poisson assumption for the marginal distributions of the V1 data. The first two tests are based on the test of the main article and test the spike count Poisson hypothesis directly. The third test is based on Kolmogorov-Smirnov plots [3] and tests a Poisson process hypothesis which is a sufficient but not a necessary condition for the spike count Poisson hypothesis. The representation of Kolmogorov-Smirnov plots is well suited to illustrate the shape of the processes.

The test of the main article can test for particular single neuron statistics as well. For this purpose Equations 10-16 together with the entropy difference as the divergence measure are applied to the Poisson distribution (Equation 20) instead of the maximum entropy distribution as the reference distribution and to the single neuron data instead of the full bivariate data. Another variant of this test can be applied to test for particular marginal distributions for bivariate data as they are considered for the original maximum entropy test. In order to restrict the test to the marginals we first shuffle the data to remove all linear and higher-order correlations. To this end, the product distribution of the Poisson marginals is used as the reference distribution and compared to the product of the empirical marginals (shuffled distribution) using entropy difference as the divergence measure. All dependencies are removed from the reference distribution and from the data, but the test is identical to the maximum entropy test otherwise.

In particular the power of this test with respect to the discrete Poisson distribution is identical to that of the proposed maximum entropy test. If a violation of the spike count Poisson assumption was the reason for the maximum entropy rejections, then we would expect rejection rates that are similar to those of the maximum entropy test. Neither the single neuron Monte Carlo goodness-of-fit test for Poisson statistics nor the multivariate Monte Carlo goodness-of-fit test for the product distribution led to any rejections of the Poisson hypothesis for the data recorded from cat V1.

The Kolmogorov-Smirnov test is based on time rescaling of the spike trains and a Kolmogorov-Smirnov plot for quantifying deviations of the interspike interval statistics from a Poisson spike generating process [3]. The interspike intervals were transformed by

$$z_k = 1 - \exp(-\lambda_i \tau_k),$$

where λ_i is the rate of the process in the time interval of the interspike interval and the τ_k are interspike intervals, corrected for discrete time by an analytical procedure of discrete time rescaling [4]. We applied the discrete time rescaling theorem [4] instead of the continuous time rescaling theorem, because neuronal spike trains were sampled with a finite sampling rate of 1 kHz. The application of the theorem removed the inhomogeneity of the processes. If the τ_k follow an exponential distribution then the z_k follow a uniform distribution. The Kolmogorov-Smirnov plots show the values of the cumulative distribution function of the uniform density defined by $b_k = \frac{k-1/2}{n}$ against the z_k values. The transformed interspike intervals of a perfect Poisson process appear as a diagonal in the Kolmogorov-Smirnov plot [3]. Confidence bounds were calculated based on a significance level $\alpha = 5\%$. The Poisson process hypothesis is rejected, if the empirical curve crosses the confidence bounds.

Spike times were discretized at 1 ms, because the spike trains were sampled with a rate of 1 kHz. Just like in the application of the maximum entropy test, we divided the spike trains into non-overlapping time intervals locked to the start of a grating presentation at varying latencies (later referred to as “time bins”). The firing rate of each bin was estimated over all trials. We then collected all interspike intervals that were confined to a single bin from each trial separately and concatenated these interspike intervals. This was done separately for the orientation of the grating stimulus, the neuron, the time bin and the two experimental conditions. For small bin sizes, interspike intervals are almost never confined to a single bin, because it is unlikely to observe more than one spike within a very small bin. We therefore used bin

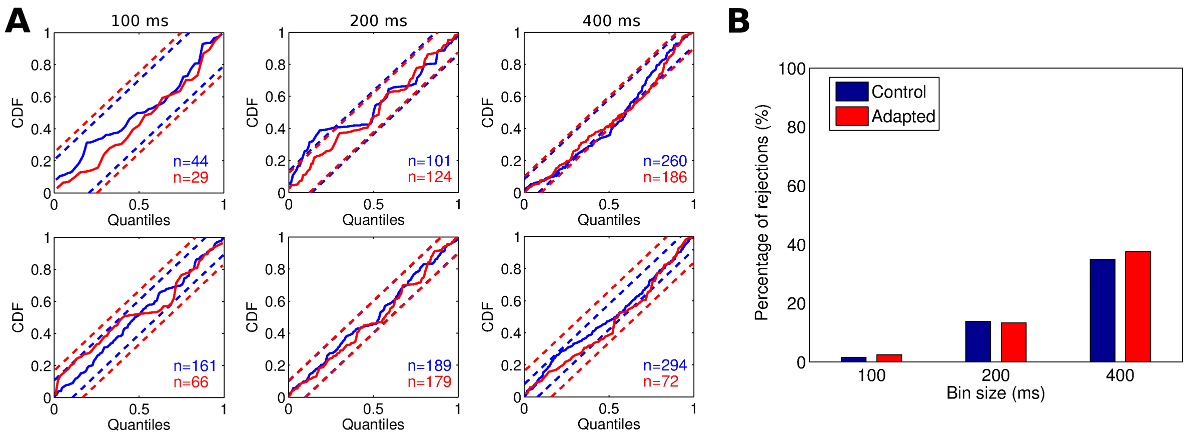

Figure S3. Kolmogorov-Smirnov tests of transformed interspike intervals. The cumulative distribution was estimated separately for neurons, stimuli, time points, control (blue) and adaptation condition (red). (A) Kolmogorov-Smirnov plots. Each subfigure corresponds to a neuron, stimulus and time bin selection. 95% confidence bounds are plotted as dashed lines. The number of samples is given in the lower right corner of each plot for the control condition (blue) and adaptation condition (red). Rates of the Poisson process were estimated for 100 ms bins (left column), 200 ms bins (center column) and 400 ms bins (right column). (B) Rejection rates of the Poisson hypothesis averaged over neurons, stimuli and time bins for different bin sizes for the control (blue) and adaptation condition (red).

sizes of sufficient length only (100 ms, 200 ms, and 400 ms) for all Kolmogorov-Smirnov plots.

Figure S3A shows a selection (orientation, time bin and neuron) of six Kolmogorov-Smirnov plots for 100 ms (left column), 200 ms (center column) and 400 ms (right column) for the control (blue) and adaptation condition (red). The closer the empirical curves are to the diagonal, the better the interspike interval statistics correspond to an ideal Poisson process. In the top center plot, the blue empirical curve crosses a confidence bound. In the top right plot, both empirical curves cross the confidence bounds. These plots, therefore, let us reject the Poisson hypotheses. The remaining plots do not let us reject the Poisson hypotheses.

The rejection rates over the complete data set of orientations, time bins and neurons for different bin sizes and conditions are shown in Figure S3B. The rates are similar for the control and adaptation condition and increase with increasing bin size. For 100 ms bins, the rejection rates are below the 5% significance level. For larger bin sizes, the rejection rates exceed the 5% significance level. The data that are rejected at smaller bin sizes are subsets of the data that are rejected at greater bin sizes, i.e. bins that are rejected at 100 ms fall into bins that are rejected at 200 ms and likewise for 200 ms and 400 ms. Note that the number of samples for the Kolmogorov-Smirnov tests increases with increasing bin size,

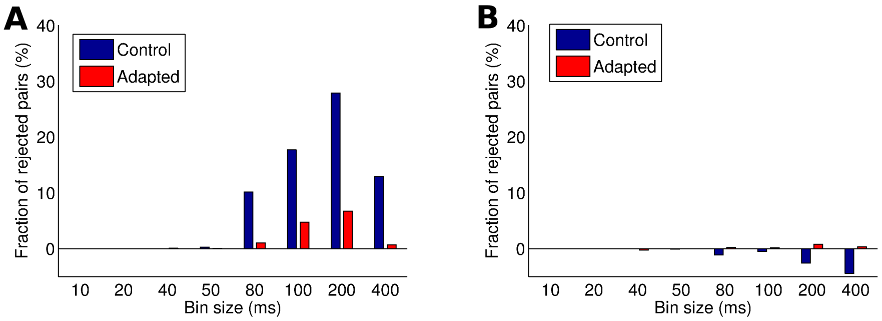

Figure S4. Results of the maximum entropy test for a subset of data recorded from area V1 of anesthetized cat that is not rejected by the Kolmogorov-Smirnov tests. Orientation, time bin and neuron pair combinations with at least one marginal Poisson rejection for 400 ms bins (cf. Figure S3B) were excluded from the analysis. (A) Fraction of neuron pairs rejected by the Monte Carlo maximum entropy test with the entropy difference as the divergence measure ($\alpha = 5\%$) and for different bin sizes. (B) Change in rejection rates compared to the full data set including data that were rejected by the Kolmogorov-Smirnov tests (cf. Figure 6A).

whereas the number of spike count samples for the maximum entropy tests do not depend on the bin size.

To investigate whether the maximum entropy rejections (cf. Figure 6) can be linked to rejections of the Poisson process hypothesis, we excluded maximum entropy rejections that were also rejected by the Kolmogorov-Smirnov tests. For an orientation and time bin, a neuron pair was excluded if a Kolmogorov-Smirnov test rejected at least one of the neurons for 400 ms bins. The rejection rates of the maximum entropy test for this subset are shown in Figure S4A. The rejection rates for the different bin sizes are similar to those for the complete data set that are shown in Figure 6A. A closer examination of the change in rejection rates that is caused by the excluded data (Figure S4B) reveals that the shift in all rejection rates is less than 5%. This result is in line with the results of the two spike count tests. We conclude that the rejections of the maximum entropy hypothesis for the entropy difference as the divergence measure cannot be explained by a violation of the Poisson process hypothesis. The change in rejection rates for the mutual information as the divergence measure was not investigated, because we would have to exclude a time bin and neuron pair whenever there was a rejection for any of the orientations.

We emphasize that the Kolmogorov-Smirnov test was applied to test a hypothesis that is a sufficient condition but not a necessary condition for the spike count Poisson hypothesis. A discrete Poisson distribution of the spike counts does not necessarily imply an underlying Poisson spike generating process. Spike counts can follow a discrete Poisson distribution even if the Kolmogorov-Smirnov test rejects the

Poisson process hypothesis entirely.

4 Alternative Marginal Distributions

There are other discrete marginal distributions that can be applied if the spike counts at hand are not Poisson distributed.

One example is the negative binomial distribution,

$$P(x;\lambda,\upsilon) = \frac{\lambda^x}{x!}\frac{1}{\left(1+\frac{\lambda}{\upsilon}\right)^{\upsilon}}\frac{\Gamma(\upsilon+x)}{\Gamma(\upsilon)(\upsilon+\lambda)^x},$$

which allows to model spike count distributions that have variance greater than mean. Here, Γ is the gamma function, λ is the mean spike count, and υ is a positive parameter which controls the degree of overdispersion. The smaller the value of υ, the greater is the Fano factor, and as υ approaches infinity, the negative binomial distribution converges to the Poisson distribution.

Another example is the binomial distribution,

$$P(x;\lambda,n) = \binom{n}{\lambda/n}(\lambda/n)^x(1-\lambda/n)^{n-x},$$

which allows to model spike count distributions that have a variance $\lambda(1-\lambda/n)$ that is smaller than the mean spike count λ. As n approaches infinity, the binomial distribution converges to the Poisson distribution.

The most appropriate distribution can be selected based on the Fano factor (defined as variance over mean). If the Fano is greater than 1 then the negative binomial distribution is promising. If it is smaller than 1 then the binomial distribution might be a better choice. If it is close to 1 then the Poisson distribution might be appropriate. These distributions can be plugged into the test procedure in place of the Poisson distribution - the only difference being one additional optimization parameter for each marginal. The distributions can also be combined: the marginal distribution of one neuron could be Poisson distributed while the marginal distribution of the other neuron is negative binomial distributed. Mixture models can be applied to construct even more complex marginal distributions in case of all three marginal distributions being rejected [5].

In principle, marginal distributions with any number of parameters can be applied in the test. Note,

however, that the required time of the simulated annealing optimization increases with the number of parameters. On current desktop computers with reasonable computation time, the number of parameters is therefore limited to less than 10 parameters per marginal distribution. In any case, empirical marginals are not feasible, because they have an unbounded number of parameters.

5 Alternative Divergence Measures

In this study, we applied the entropy difference and the mutual information difference as divergence measures. The proposed maximum entropy test, however, is more general and makes it possible to apply other divergence measures D as well. In the following, we list a couple of alternative divergence measures.

A very commonly applied divergence measure in statistical testing is Pearson's X^2 statistic

$$D_{X^2}(P^{(1)}, P^{(2)}) = \sum_{\boldsymbol{x}} \frac{(P^{(1)}(\boldsymbol{x}) - P^{(2)}(\boldsymbol{x}))^2}{P^{(2)}(\boldsymbol{x})},$$

where $P^{(1)}$ is the empirical distribution and $P^{(2)}$ is the reference distribution (also called null distribution). This divergence measure is primarily motivated by its asymptotic characteristics. The statistic is asymptotically χ^2-distributed when the number of samples approaches infinity [6]. In the proposed Monte Carlo test the asymptotic distribution is of no concern, because the test is specifically designed to be applicable when the number of samples is small. Nevertheless, the statistic quantifies the distribution difference on a point-by-point basis. The X^2 statistic is part of the power divergence family of goodness-of-fit statistics [7] which provides many choices for divergence measures.

The Kullback-Leibler divergence (KL divergence) is a natural choice for quantifying divergence between distributions:

$$D_{KL}(P^{(1)}, P^{(2)}) = \sum_{\boldsymbol{x}} P^{(1)}(\boldsymbol{x}) \log \left(\frac{P^{(1)}(\boldsymbol{x})}{P^{(2)}(\boldsymbol{x})} \right).$$

KL divergence can be interpreted as the expected extra message length per data point that must be communicated if a code that is optimal for a given distribution $P^{(2)}$ instead of a true distribution $P^{(1)}$ is used [8]. The measure is also related to the mutual information by

$$I(X;Y) = D_{KL}(P(X,Y), P(X)P(Y)).$$

The divergence measure quantifies the divergence between two distributions. Any function that quantifies such a divergence can be applied in the test. The test then assesses the necessity of higher-order correlations in terms of the divergence measure. The choice of this measure should therefore depend on the task at hand. If the task is to evaluate the necessity of higher-order correlations in terms of the mutual information, then the divergence measure should also be based on the mutual information.

6 Modeling Higher-order Correlations

Suppose that the right marginal distributions were selected and that the maximum entropy hypothesis is rejected by the proposed test. In that case, a model that includes higher-order correlations is required. Generalized linear models (GLMs) have become very popular in the neuroscience literature [9–12] and include higher-order dependencies. Here, we will briefly describe the basic framework of GLMs and relate it to the maximum entropy models which we applied in the proposed test.

In the GLM framework for spike trains, spiking statistics are based on conditional intensity functions. For two coupled neurons Y and Z, the following conditional intensity functions are defined [12]:

$$\begin{aligned} \lambda_{yt} &= \exp(\boldsymbol{k}_y \cdot \boldsymbol{x}_t + \boldsymbol{h}_{yy} \cdot \boldsymbol{y}_{[t-\tau,t)} + \boldsymbol{h}_{yz} \cdot \boldsymbol{z}_{[t-\tau,t)}) \\ \lambda_{zt} &= \exp(\boldsymbol{k}_z \cdot \boldsymbol{x}_t + \boldsymbol{h}_{zz} \cdot \boldsymbol{z}_{[t-\tau,t)} + \boldsymbol{h}_{zy} \cdot \boldsymbol{y}_{[t-\tau,t)}), \end{aligned}$$

where $\boldsymbol{k}_y, \boldsymbol{k}_z, \boldsymbol{h}_{yy}, \boldsymbol{h}_{zz}, \boldsymbol{h}_{yz}$ and $\boldsymbol{h}_{zy}$ are filters, $\cdot$ denotes the dot product and $\boldsymbol{h} \cdot \boldsymbol{y}_{[t-\tau,t)} = \sum_{i=t-\tau}^{t-\Delta t} h_i y_i$ denotes the filtered activity of Y in the time interval $[t-\tau, t)$. The distributions $P(y_t|\lambda_{yt})$ and $P(z_t|\lambda_{zt})$ of the activities conditioned on the intensity functions can, for instance, be Poisson or Bernoulli distributed [10]. Conditional independence of these distributions is assumed. Thus, the full likelihood function of the model is given by

$$P(Y, Z|X, \boldsymbol{k}_y, \boldsymbol{k}_z, \boldsymbol{h}_{yy}, \boldsymbol{h}_{zz}, \boldsymbol{h}_{yz}, \boldsymbol{h}_{zy}) = \prod_t P(y_t|\lambda_{yt}) P(z_t|\lambda_{zt}).$$

$\boldsymbol{k}_y, \boldsymbol{h}_{yy}$ and $\boldsymbol{h}_{yz}$ are referred to as stimulus filter, history filter and coupling filter of neuron Y, respectively. Typically, these filters are parametrized by basis functions like, for instance, cosine bumps [11]. The coupling filters introduce dependencies between the neurons. Even more complex dependencies can

be modeled by hidden neurons [12]. Note, however, that changing the dependencies also changes the marginal distributions of the neurons. There are efficient fitting procedures for the parameters of the model [11]. Moreover, GLMs can model non-stationary data by their history dependent terms. For a more detailed description of GLMs we refer the reader to previous studies [9–12].

In contrast to GLMs, the second-order maximum entropy models that we apply in the test do not have history dependence. Instead, separate models are generated for each point in time. The models therefore do not make any assumptions about non-stationarity which could be confounded with effects of higher-order dependencies. History effects within a single spike count bin can be modeled by the marginal distributions. If there are autocorrelations present then the marginal spike count distributions will typically deviate from discrete Poisson distributions (cf. Section "Impact of Autocorrelations" of the main article). We describe alternative marginal distributions in Section "Alternative Marginal Distributions" for modeling such data.

7 Subpopulation Structure of Recorded Neurons

The proposed maximum entropy test provides a method to check whether significant divergences between the second-order model and data are present, but it does not provide quantitative differences. To get an idea of the magnitude of these differences we applied two estimators. (1) An estimator that is based on the second-order model. We used the maximum likelihood second-order maximum entropy model and calculated the mutual information based on this distribution. (2) A state-of-the-art bias correction estimator working directly on the data. Here we applied the shuffle estimator [13] together with the best upper bound (BUB) estimator [14]. The BUB estimator does not rely on the asymptotic sampling regime and therefore might work in the undersampled regime. In practice, however, the number of samples in each condition should be larger than the number of relevant responses in order to obtain an unbiased estimate of the mutual information [15]. For small Poisson rates where the domain can be restricted to 15 spike counts the number of relevant responses is 225. Thus, at least 225 samples per stimulus are required for an unbiased estimate, whereas only 42 samples per stimulus are present in our data set. Nevertheless, the relation between the bias correction estimator and the second-order maximum entropy estimator may illustrate the order of the impact of higher-order correlations.

The maximum entropy test examines the sufficiency of the second-order model in terms of the mutual

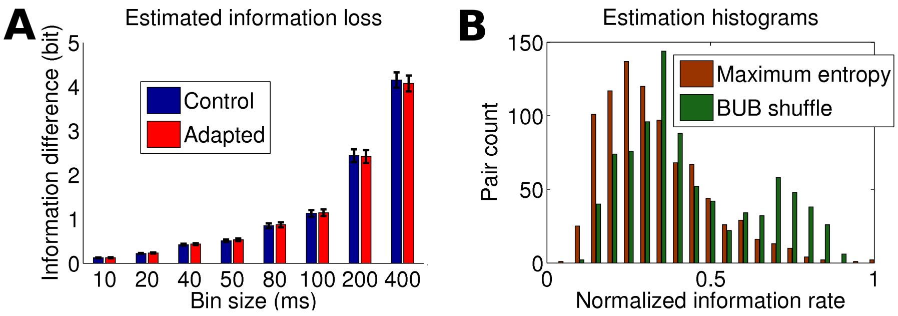

Figure S5. Pairwise mutual information estimates. The mutual information was averaged over all pairs and subsequent bins. (A) Difference between the mutual information estimated by the maximum entropy distribution and the (higher) mutual information estimated by the BUB shuffle estimator for the same pairs. (B) Histogram over the information rate estimated by the maximum entropy distribution and the information rate estimated by the mutual information BUB shuffle estimator. The information rates are normalized to the respective maximum information rate of the estimator. Control and adaptation conditions are mixed.

information difference. Therefore, we expected the differences of the mutual information estimations to reflect the rejection rates of the maximum entropy test. The differences between estimator 1 and estimator 2 are shown in Figure S5A. For all but the 200 ms and 400 ms bins the differences between the estimates are smaller for the control condition than for the adaptation condition. Differences between control and adapted condition are not significant though. The total differences between the mutual information estimates are on the order of 0.3 to 4 bit. This amounts to almost two times the mutual information of the second-order model and suggests a strong impact of higher-order correlations on the mutual information.

We tested all pairs of the recorded neurons but there could be subpopulations of neurons with strong higher-order correlations. To investigate whether this is the case we examined the empirical distributions of the information rates. Histograms over the information rates of the maximum entropy estimator and of the BUB shuffle estimator are shown in Figure S5B. We normalized the information rates to their respective maximum to make the distributions easily comparable. The distribution of the BUB shuffle estimator has a remarkable bimodal form which suggests subpopulations with (1) low and (2) high pairwise mutual information. We applied Hartigan's dip test of unimodality [16] with 500 bootstrap samples in order to verify the multimodal form of the latter distribution. The hypothesis of unimodality can be rejected for the distribution of the BUB shuffle estimator ($p < 1\%$), whereas it cannot be rejected for the distribution of the maximum entropy estimator ($p = 77.6\%$). The recorded neurons are presented

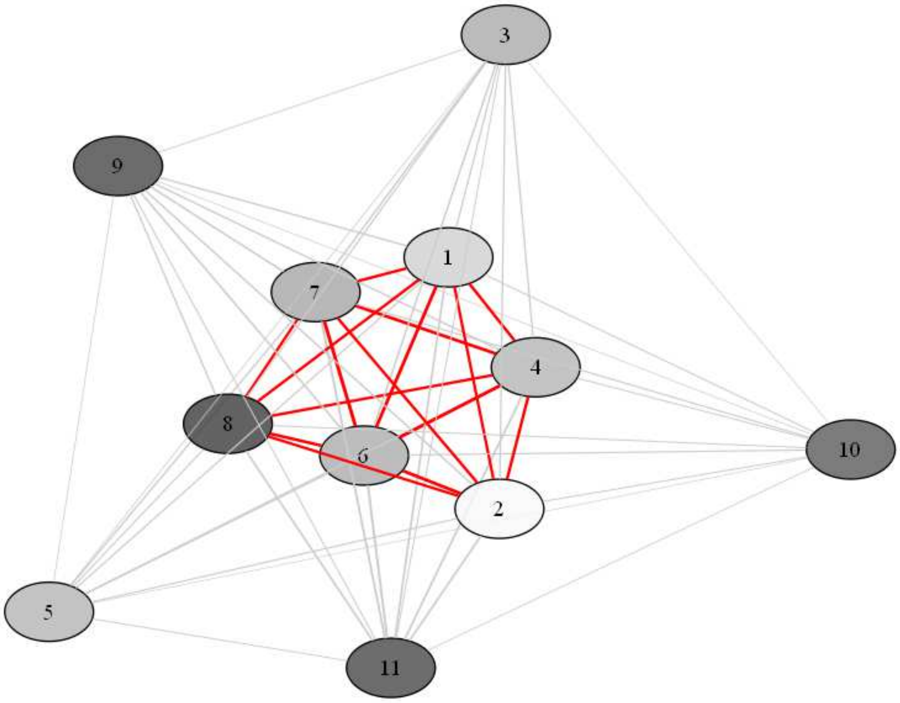

Figure S6. The graph of the neurons that were recorded from anesthetized cat V1 during an adaptation experiment. The nodes represent the neurons. The numbers in the nodes are the indices of the neurons. Gray values visualize preferred orientations of the cells. Edges are shown in red if the information rate of the pair as estimated by the BUB shuffle estimator exceeds 1.7 bit/s.

as a graph in Figure S6. The edges of group 2 are shown in red. All neurons in this group (1, 2, 4, 6, 7 and 8) are connected by red lines and thus form a subpopulation with high pairwise mutual information. This separation coincides with a thresholding of the overall firing rate of the individual neurons at 10 Hz (Figure 7A of the main article).

References

1. Horst R, Pardalos PM (1995) Handbook of Global Optimization. Dordrecht (Netherlands): Kluver.

2. Kirkpatrick S, Gelatt CD, Vecchi MP (1983) Optimization by simulated annealing. Science 220: 671–680.

3. Brown EN, Barbieri R, Ventura V, Kass RE, Frank LM (2001) The time-rescaling theorem and its application to neural spike train data analysis. Neural Comput 14: 325–346.

4. Haslinger R, Pipa G, Brown E (2010) Discrete time rescaling theorem: determining goodness of fit for discrete time statistical models of neural spiking. Neural Comput 22: 2477–2506.

5. Everitt BS, Hand DJ (1981) Finite mixture distributions. London: Chapman and Hall.

6. Cochran WG (1952) The χ^2 test of goodness of fit. Ann Math Stat 23: 315–345.

7. Cressie N, Read TRC (1989) Pearson's X^2 and the loglikelihood ratio statistic G^2: a comparative review. Int Stat Rev 57: 19–43.

8. Cover TM, Thomas JA (1991) Elements of information theory. New York: Wiley.

9. Kass RE, Ventura V (2001) A spike-train probability model. Neural Comput 13: 1713–1720.

10. Truccolo W, Eden UT, Fellows MR, Donoghue JP, Brown EN (2005) A point process framework for relating neural spiking activity to spiking history, neural ensemble, and extrinsic covariate effects. J Neurophysiol 93: 1074–1089.

11. Pillow JW, Shlens J, Paninski L, Sher A, Litke AM, et al. (2008) Spatio-temporal correlations and visual signalling in a complete neuronal population. Nature 454: 995–999.

12. Pillow J, Latham P (2008) Neural characterization in partially observed populations of spiking neurons. In: Platt JC, Koller D, Singer Y, Roweis S, editors, Advances in Neural Information Processing Systems 20, Cambridge, MA: MIT Press. pp. 1161–1168.

13. Montemurro MA, Senatore R, Panzeri S (2007) Tight data-robust bounds to mutual information combining shuffling and model selection techniques. Neural Comput 19: 2913–2957.

14. Paninski L (2003) Estimation of entropy and mutual information. Neural Comput 15: 1191–1253.

15. Panzeri S, Senatore R, Montemurro MA, Petersen RS (2007) Correcting for the sampling bias problem in spike train information measures. J Neurophysiol 98: 1064–1072.

16. Hartigan JA, Hartigan PM (1985) The dip test of unimodality. Ann Stat 13: 70–84.
